# Virtual HIV pre-exposure prophylaxis outpatient service in the era of COVID-19

**DOI:** 10.1177/0956462420961951

**Published:** 2020-10-13

**Authors:** Siobhan Quirke, Laura Quinn, Deborah Hegarty, Aisling Loy, Fiona Lyons, Fiona Mulcahy, Emma Devitt

**Affiliations:** Department of Genitourinary Medicine & Infectious Diseases (GUIDe), St James’s Hospital, Dublin, Ireland

**Keywords:** Human immunodeficiency virus, prevention, antiretroviral therapy

## Abstract

In the midst of the COVID-19 pandemic, health care providers have had to rapidly change how they deliver care to patients. We discuss how we are delivering a virtual HIV pre-exposure prophylaxis (PrEP) service during this time; challenges faced; challenges expected and goals for the coming months.

## Background

On the 24th March 2020 the Irish government announced the lockdown of businesses, venues, facilities and amenities. Three days later all non-essential travel and contact with those outside one’s home was prohibited. Since these restrictions were announced the COVID-19 pandemic has led to radical changes in the delivery of healthcare in Ireland. Over a number of weeks hospitals were forced to develop policies and revise current processes of healthcare delivery in order to prepare for the expected surge of patients with COVID-19, to comply with public health social distancing advice and to safeguard patients and staff. This involved the cancellation of thousands of procedures and outpatient appointments but also changing the delivery of outpatient services to a virtual platform where possible, as recommended by the HSE and Public Health Emergency Team.

PrEP is the preemptive use of oral antiretroviral therapy (tenofovir (TDF) and emtricitabine (FTC) by HIV-negative individuals to reduce the risk of HIV infection. It has been found to be safe and highly effective at preventing HIV infection in those at substantial risk if used as instructed. In November 2019, the Irish Health Service Executive (HSE) commenced free HIV pre-exposure prophylaxis (PrEP) for those meeting clinical eligibility criteria and deemed at substantial risk of HIV infection.^1^,^2^

The GUIDE clinic at St James’s Hospital, Dublin is the largest HIV and STI service in Ireland. A new HIV prevention/PrEP service was established here in November 2019 to deliver publicly funded PrEP in two weekly clinics. This involved approximately 30 attendances to our PrEP service here each week. Prior to the COVID-19 pandemic patients could access the clinic either by self-referral through online booking or provider referral via existing general sexual health clinics (eg. PEP (post exposure prophylaxis) to PrEP transition).

Until COVID-19 lockdown in March 2020 we offered a face-to-face initial assessment. This involved taking details of medical history, sexual history and drug history. Baseline bloods would be taken as well as an STI screen and a urinary PCR (uPCR). Safe sexual practices were discussed and vaccination needs were addressed (HPV, Hepatitis A & B). A comprehensive HIV risk assessment would be performed and risk reduction measures discussed. There are referral pathways in place to local ‘Chemsex’/Club Drug Clinic services as required, these services are still available but are modified in line with public health advice.^3^ Individuals would be counseled regarding use of PrEP (side effects, importance of adherence, dosing schedules) and provided with a three month prescription. In Ireland PrEP medication is dispensed by community pharmacies to those who are registered as eligible for the publicly funded drug. In order to access this an individual must be attending a designated PrEP service for monitoring and ongoing prescribing and registered on the Primary Care Reimbursement System (PCRS) by the PrEP prescriber.

Patients are reviewed at three monthly intervals in line with national guidelines and repeat HIV testing, renal monitoring and STI screen are performed.^1^,^2^

Our cohort includes 387 patients (November 2019 - August 2020) and is majority Caucasian, non-Irish born cismale and majority men who have sex with men (MSM) (Table 1).

**Table 1.** Cohort characteristics

Gender	Number of patients	Percentage of cohort
Male	385	99.5
Female	0	0
Trans Female	2	0.5
Age – Median in Years (Range)		
32 (18–75)		
Ethnicity		
White - Irish	176	45.5
White - Other	70	18
Roma	1	0.2
Other incl Mixed Background	81	20.9
Black/Black Irish	1	0.2
Asian/Asian-Irish	16	4.13
Not documented	42	10.8
Sexual Orientation		
Heterosexual (with casual male partners)	6	1.5
Bisexual	53	13.7
MSM	328	87.6
Drug Use		
Chemsex (within past 6 months)	65	16.9%

## Converting to a virtual platform

With the arrival and surge of COVID-19 in Ireland virtual PrEP clinics began in our department on the 18th March 2020 in preparation for the pending nationwide lockdown. On the 23rd March 2020 the majority of GUIDE clinic staff were redeployed to frontline services. At this point the clinic was closed and the HIV and PrEP services continued on a virtual platform with the goals of reducing the number of people attending the clinic thus reducing exposure to patients and to staff whilst maintaining HIV care and HIV prevention service delivery. Additionally, with the mandated restriction on public movement we expected to see a drop in rates of new STI diagnoses and in those seeking PEP and PrEP due to reduced sexual activity outside the home. The clinic website was updated to reflect changes made to the service and patients with pre-booked appointments received a text message updating them that the clinic was closed.^4^

During the initial phase of lockdown, from the 18th March to 2nd June 2020, our virtual clinic involved advance consultant review of the electronic medical records and co-prescribed medications of the patients booked in to that clinic. Phone calls were made to patients as required, prioritising those with comorbidities or who were felt to be more vulnerable. In particular, patients with known renal impairment were contacted for virtual review. Due to uncertainty at the time a 6 month PrEP prescription was provided in case further restrictions on movement were implemented or a second surge of COVID-19 occurred, but patients were given a 3 monthly review appointment as recommended in the National Guidelines.^1^,^2^ Written prescriptions (for TDF/FTC) were posted to each patient by mail, the patient was registered as usual on the Primary Care Reimbursement System (PCRS) and they attended a local community pharmacy to collect the medication.

The decision to continue PrEP in the absence of blood testing in the initial phase of lockdown in those at ongoing risk of HIV was made due to high effectiveness of PrEP at HIV prevention and low risk of drug toxicity in the majority of individuals.

On the 18th May 2020 Ireland entered Phase 1, the first step of the government plan to ease COVID-19 restrictions and reopen the country.^5^ Soon after this initial phase of reopening some staff returned to the clinic and the new PrEP service commenced on the 2nd June 2020. Our PrEP service evolved to a phone consultation by the Clinical Nurse Specialist for all new and returning PrEP patients. After the phone consultation patients are asked to attend the clinic for rapid STI screening and bloods. This minimises the time spent on site and the amount of face-to-face contact while allowing the usual care to proceed. They are all booked for a 3 month follow up review. The plans for how this review appointment will take place are evolving with public health advice but it is likely that phone consultation followed by rapid STI and blood testing will continue. Local lockdowns commenced in August 2020 and restrictions on travel for some patients mean the clinic model will adapt to the patients needs at the time of their scheduled review. In person review with a doctor (as per the pre-COVID model) is available in a weekly clinic for high risk individuals with language barriers, comorbidities, psychosocial issues or other concerns.

Those with symptomatic STIs were and continue to be triaged by our phone service and seen for emergency assessment and treatment as required.

Now, as outpatient clinics have reopened and all staff have returned from frontline services *all* patients are receiving a phone clinic review by the Clinical Nurse Specialist. Since the 2nd June there have been 227 patients contacted, virtually assessed and asked to attend for rapid appointment, 19 new patients commenced on PrEP and 11 have transitioned from PEP to PrEP. Routine HIV testing and other STI testing have recommenced and our clinic proformas have been adapted appropriately for virtual review. Additionally, staff have been trained in the use of new equipment (second computer screens, headsets), clinic templates and updated clinic codes to capture activity.

We have had to stop self-referral online booking due to capacity constraints from staff absences and to comply with social distancing measures in the department; this is due to reopen in September 2020. Due to ongoing national guidance to stay at home, limited access to social outlets and widespread social distancing, the number of individuals seeking PEP/PrEP has dramatically reduced during the pandemic. A small number of our patients discontinued PrEP themselves during the lockdown period, as they were not at risk of HIV during this time and did not wish to take unnecessary medication. These patients have recommenced PrEP either themselves as they resumed sexual activity outside the home or since the reopening of the PrEP service. Others switched themselves from daily dosing to event based dosing as required.

## Benefits and challenges

Telemedicine and virtual clinics have been described by many as a silver lining in the midst of this global pandemic. The benefits of switching to virtual review are clear. Patients can access specialist care while limiting risks of exposure to and spread of COVID-19 in the hospital/clinic setting. A number of queries and issues can be dealt with remotely and clinical needs can be triaged. It enabled this service to continue with reduced staff numbers due to redeployment. It has lower costs and is a convenient option for those who have access to email/smart phones.^6^

However, while there are a number of benefits it is not without significant challenges and limitations. Prior to the SARS-CoV-2 pandemic telemedicine played a very small role in our day to day tertiary level activity. It can be time-consuming with some patients requiring multiple phone calls in order to get through to them for consultation. Digital inequalities have been well described amongst the elderly population when it comes to telemedicine, this was not an issue that we faced as all our patients have access to a phone/smartphone. Particular challenges we have faced with our patient cohort include frequent changing of address or phone number. In the vast majority of cases we successfully contacted each patient with either letter or phone correspondence. Additionally, language barrier is a limitation faced by phone consultation, particularly with new patients.

During lockdown there has been a dramatic reduction in face to face clinical assessments, there has also been an impact on PEP, PrEP and sexual behaviour globally. In recent weeks there has been updated data published on changes in post-exposure prophylaxis after sexual exposure (PEPSE) during COVID-19 lockdown. 56 Dean Street, a sexual health clinic in Soho, London has since published a paper outlining their experience of this. They found that there was more than an 80% reduction in PEPSE prescriptions in the first four weeks of lockdown in the UK (23rd March - 19th April 2020) compared to the four weeks immediately preceding lockdown.^7^,^8^ We had very similar findings on comparison of the same time period in our department, we recorded a 78% drop in PEP prescriptions, with 7 patients receiving prescriptions in the first four weeks of lockdown compared to 31 in the preceding four weeks.

While we noted only a few patients discontinued PrEP during lockdown this impact has been reflected in some recent studies from Australia and the United States. Hammoud M et al. in Sydney looked at the impact of physical distancing due to COVID-19 in a group of 940 gay and bisexual men. 46% of this group had been on PrEP prior to lockdown and 58% of this group continued to use it. Sexual activity was reduced by 50–60%. Of those who stopped taking PrEP 86% stopped due to COVID-19 while 17% said they could not access PrEP services during this time.^9^ Brawley S et al. in the United States demonstrated similar themes of change in sexual behaviour and reduced PrEP uptake with a reduction in daily and event based usage. In terms of service provision this study found that 68% of services provided phone consultations/telemedicine for some appointments, 43% offered virtual appointments only, 22% had limited services and 2.6% had stopped providing PrEP in any capacity.^10^

While we have succeeded in maintaining continuation of HIV PrEP in those already attending the service, deferral of services for new patients, particularly in the initial phase of lockdown means that we have potentially missed new asymptomatic infections. At present, Ireland does not have freely available home STI testing. A pilot programme is planned but owing to COVID-19 has been delayed. There is a notable campaign in London from 56 Dean Street clinic advocating widespread home HIV testing during the lockdown when transmission is likely to be low due to social distancing.^7^ In theory if all individuals who had HIV risk pre lockdown were to test prior to the lifting of restrictions and engage with rapid start antiretroviral treatment (or PrEP if negative) post COVID HIV transmission could be reduced.

We are aware that while the use of telemedicine and virtual review can be beneficial from a health care provider perspective. This may not be the experience of the patient. We plan to evaluate patient satisfaction with the service with an online feedback questionnaire in the coming months. To date patient engagement and verbal feedback has been overwhelmingly positive.

## The way forward

The phased reopening of our country has begun and with this comes the reinstatement of outpatient appointments and elective procedures. Regarding our PrEP clinic there are a number of considerations for the coming weeks and months.

In the past number of weeks, as staff have returned to clinics from the frontline rapid appointments have been given to patients for self testing and bloods. We have prioritised and encouraged patients who have not received recent testing to attend. Unfortunately there are no home testing kits available in Ireland as of yet.

Our aim is to have the self-referral and online booking system running again by September 2020, however this will require new patient flow in the clinic to adhere to social distancing requirements and revised booking templates. It is likely that face to face clinics will be reduced long term in favour of combined telephone consultation and self taken STI testing. We have developed a patient information leaflet outlining the changes to the service and regularly update our website.^4^

In the interim clinic models include the following:

- Continuing virtual clinic phone review with patients to determine whether they are eligible for PrEP, then asking them to attend a rapid appointment for pre booked bloods/STI screen, vaccination and prescription collection
- Online medical triage form to be completed by the patient prior to clinic appointment

The way forward will not be without challenge and uncertainty but for now virtual practice allows us to continue to provide this service to the community.
